# AMG-232 sensitizes high MDM2-expressing tumor cells to T-cell-mediated killing

**DOI:** 10.1038/s41420-020-0292-1

**Published:** 2020-07-06

**Authors:** Ilyas Sahin, Shengliang Zhang, Arunasalam Navaraj, Lanlan Zhou, Don Dizon, Howard Safran, Wafik S. El-Deiry

**Affiliations:** 1 Joint Program in Cancer Biology, Brown University and Lifespan Health System, Providence, RI USA; 2 Division of Hematology/Oncology, The Warren Alpert Medical School, Brown University, Providence, RI USA; 3 Department of Pathology & Laboratory Medicine, The Warren Alpert Medical School, Brown University, Providence, RI USA; 4 Cancer Center at Brown University, The Warren Alpert Medical School, Brown University, Providence, RI USA

**Keywords:** Cancer immunotherapy, Drug development

## Abstract

Oncogenic mouse double minute 2 homolog (MDM2) is an E3-ubiquitin ligase that facilitates proteasomal degradation of p53. MDM2 amplification occurs in cancer and has been implicated in accelerated tumor growth, known as hyper-progression, following immune-checkpoint therapy. MDM2 amplification also predicts poor response to immune-checkpoint inhibitors. We sought to evaluate the role of MDM2 in T-cell-mediated immune resistance. Ovarian clear cell carcinoma cell lines carrying wild-type p53 with low/high MDM2 expression were investigated in a T-cell co-culture system evaluating T-cell-mediated tumor killing. Targeting of MDM2 was achieved by siRNA transfection or a selective MDM2 inhibitor, AMG-232 and tumor cells were tested in the T-cell co-culture system. AMG-232 activated p53 signaling in cancer cells and relative resistance to AMG-232 was observed in high MDM2-expressing cell lines. Cell lines with high MDM2 expression were more resistant to T cell-mediated tumor killing. Targeting MDM2 by gene-silencing or pharmacological blockade with AMG-232 enhanced T-cell killing of cancer cells. AMG-232 potentiated tumor cell killing by T-cells in combination with anti-PD-1 antibody treatment, regardless of changes in PD-L1 expression. The AMG-232 was not toxic to the T-cells. MDM2 inhibition lowered expression of Interleukin-6, a pro-inflammatory pro-tumorigenic cytokine. Our data support targeting MDM2 in tumors with overexpression or amplification of MDM2 as a precision therapy approach to overcome drug resistance including hyper-progression in the context of immune checkpoint therapy.

## Introduction

Immune checkpoint inhibitors (ICIs) have revolutionized cancer treatment with significant responses for a variety of previously refractory tumors. However, primary or acquired therapy resistance in about two-thirds of patients receiving ICIs remains as a major challenge^1^. In addition to primary resistance, another challenge is the phenomenon known as hyper-progressive disease (HPD), i.e. the acceleration of tumor growth after ICIs observed in 5–29% of patients. HPD has recently been described with different immune-checkpoint blocking agents for different tumor types including, but not limited to, breast, head and neck, gastric, genitourinary and non‐small-cell lung cancers^2^. Amplification of the mouse double minute 2 homolog (MDM2), an E3-ubiquitin protein ligase that facilitates proteasomal degradation of p53, has been suggested as a recurring genomic correlate of increased risk of HPD in clinical studies^3,4^. A recent study with a cohort of 1105 patients obtained from MSK-IMPACT Clinical Sequencing Cohort (MSKCC) suggested that MDM2/4 amplification predicts poor response to ICIs in a variety of cancers^5^. Taken together, these findings provided a strong rationale for exploration of targeting MDM2 as a potential strategy to overcome ICI-resistance or HPD. We found that high MDM2-expressing ovarian cancer cell lines are more resistant to T-cell-mediated killing and can be sensitized through targeting MDM2 by siRNA or pharmacological inhibition with AMG-232. Our findings provide preclinical evidence for targeting MDM2 as a reasonable approach to overcome T-cell-mediated immune resistance or HPD and to design more effective cancer immunotherapy strategies.

## Results

### High MDM2 expression contributes to immune evasion by cancer cells

**Fig. 1:**
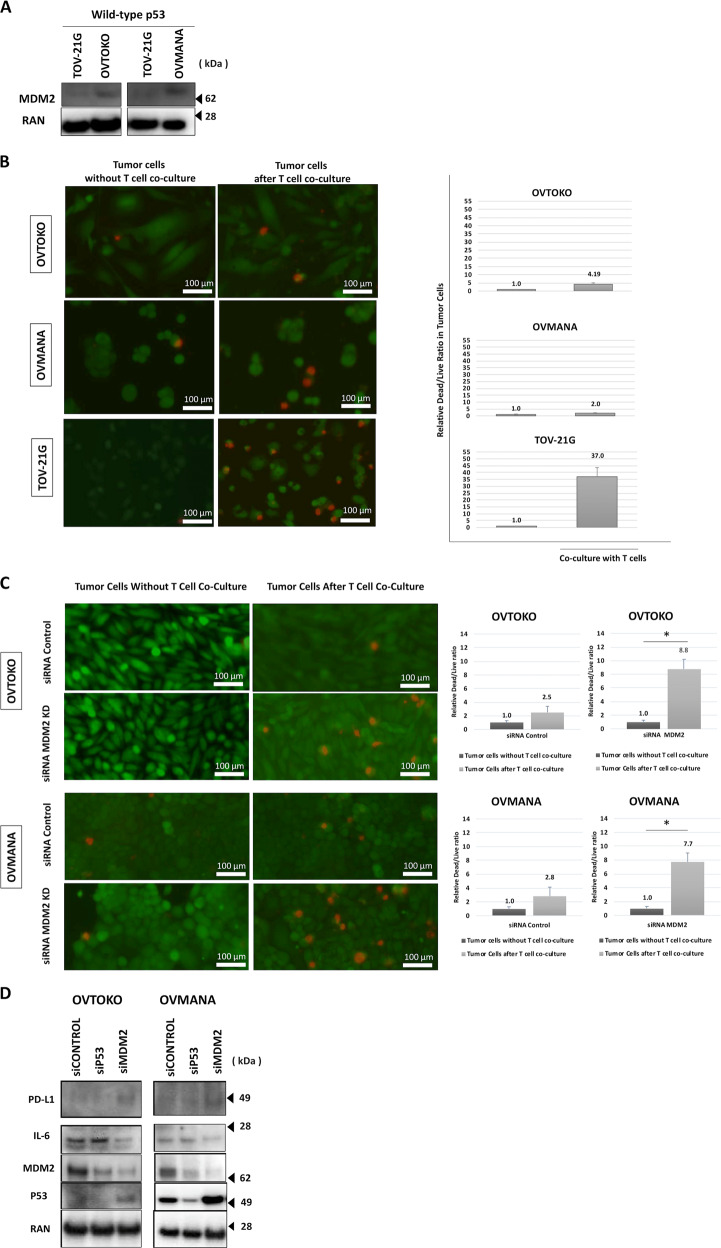
High MDM2 expression contributes to immune evasion. **a** MDM2 expression in ovarian clear cell carcinoma cell lines (OVTOKO, OVMANA, and TOV-21G) carrying p53 wild-type was assessed by immunoblotting. **b** Green-fluorescent tumor cells were co-cultured with cytotoxic T-cells (TALL-104) with a 2:1 effector-to target cell ratio (E:T) for 12 h. Red-fluorescent ethidium homodimer-1 was used to detect dead cells. Representative images of live/dead tumor cells for each cell line are shown (left panel). Random fields were analyzed, and dead/live cells were quantified by immunofluorescence (right panel). Scale bars, 100 μm. **c** Similarly, the effect of silencing MDM2 by siRNA in high MDM2-expressing cell lines (OVTOKO and OVMANA) on T-cell-mediated killing were tested. Scale bars, 100 μm. **d** Confirmation of MDM2 knockdown and the effects of si-p53 and si-MDM2 on IL-6, PD-L1, and p53 expression were assessed by immunoblotting.

We examined MDM2 expression in a panel of three wild-type p53-expressing human ovarian clear cell carcinoma cell lines and found higher expression of MDM2 (OVTOKO and OVMANA) as compared to TOV-21G (Fig. 1a). The MDM2 expression profile is consistent with a previous report^6^. We next performed T-cell co-culture experiments with a 2:1 effector-to-target (E:T) cell ratio for 12 h. Tumor cells were co-cultured with interleukin-2 (IL-2) activated pre-treated cytotoxic TAL-104 T-cells to evaluate T-cell-mediated killing via quantification of dead/live tumor cells. After T-cell co-culture, TOV-21G tumor cells were found to have a dead/live tumor cell ratio of 37:1 as compared to tumor cells alone (*P* = 0.01), whereas high MDM2-expressing tumor cells (OVTOKO and OVMANA) were found to be more resistant to T-cell killing with a much lower ratio of dead/live tumor cells compared to tumor cells alone (4.2:1 and 1.9:1; *P* = 0.03 and *P* = 0.07, respectively; Fig. 1b). Supplementary Video 1 shows a representative video of OVTOKO and TOV-21G with and without T-cell co-culture in the presence of EthD-1 which fluorescently labels dead cells, further illustrating these differential T-cell killing effects associated with MDM2 expression.

To test the effect of silencing MDM2 on T-cell-mediated tumor killing, MDM2- or control-siRNA transfected OVTOKO and OVMANA tumor cells were examined after T-cell co-culture. Using tumor cells alone as an internal control, silencing MDM2 in both cell lines showed a significant increase in dead/live tumor cell ratio after 12 h of T-cell co-culture (Fig. 1c). OVTOKO and OVMANA MDM2-siRNA tumor cells compared to tumor cells alone had a dead/live tumor cell ratio of 8.8:1 (*P* = 0.03) and 7.7:1 (*P* = 0.03), respectively. Supplementary Video 2 shows a representative video of control- or MDM2-siRNA transfected OVTOKO and OVMANA cell lines in the T-cell co-culture system in the presence of EthD-1 which indicates dead cells by their fluorescent red labeling. On the other hand, OVTOKO and OVMANA siRNA control cells as compared to tumor cells alone had a dead/live tumor cell ratio of 2.5:1 (*P* = 0.2) and 2.8:1 (*P* = 0.3), respectively. The efficiency of knockdown for both cell lines was confirmed by immunoblotting (Fig. 1d). Importantly, MDM2-knockdown cells showed reduction in interleukin-6 (IL-6), a proinflammatory cytokine that promotes tumorigenesis by multiple signaling pathways, in both cell lines. Baseline PD-L1 protein expression was found to be low and a slight increase in PD-L1 expression with MDM2 knockdown was observed.

### Targeting MDM2 by AMG-232 sensitizes high MDM2-expressing tumor cells to T-cell-mediated killing

**Fig. 2:**
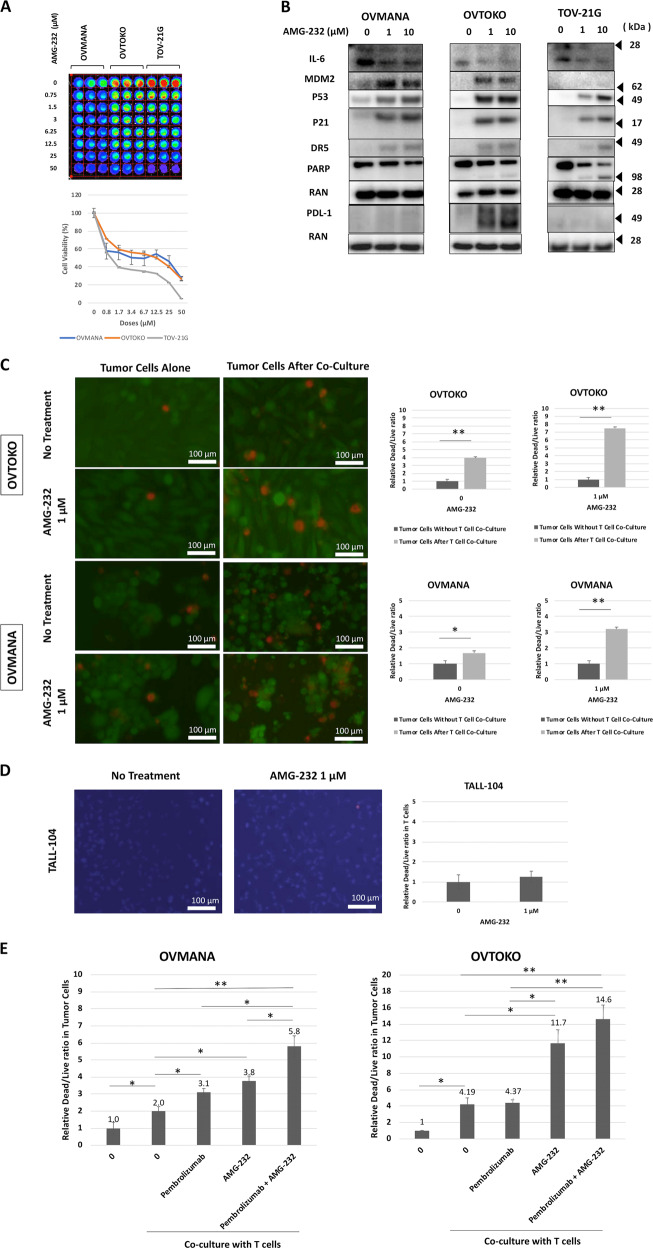
Targeting MDM2 by AMG-232 sensitizes high MDM2-expressing tumor cells to T-cell-mediated killing. **a** Cell lines were treated with different doses of AMG-232 for 48 h and cell viability was assessed by using the CellTiter-Glo assay (upper panel with image, lower panel with quantitative analysis). **b** The effect of AMG-232 on the p53 signaling pathway, and the expression of IL-6 and PD-L1 at different drug doses was assessed by immunoblotting. **c** Green-fluorescent tumor cells were pre-treated with or without 1 µM AMG-232 and co-cultured with cytotoxic T-cells (E:T; 2:1) for another 16 h with or without 1 µM AMG-232. Red-fluorescent ethidium homodimer-1 was used to detect dead cells. Representative images of live/dead tumor cells are shown (left panel). Random fields were analyzed, and dead/live cells were quantified by immunofluorescence (right panel). Scale bars, 100 μm. **d** Similarly, blue-fluorescent T-cells were treated with or without AMG-232 and red-fluorescent ethidium homodimer-1 was used to detect dead T-cells. Scale bars, 100 μm. **e** Tumor cells were co-cultured with cytotoxic T-cells (E:T; 2:1) for 16 h with and without 1 µM AMG-232 and 25 µg/mL of the anti-PD-1 antibody pembrolizumab. Random fields were analyzed, and dead/live cells were quantified by immunofluorescence.

Based on the MDM2-knockdown data, we further investigated the potential strategy for targeting MDM2 through pharmacological inhibition of MDM2 by AMG-232, an investigational orally-bioavailable selective MDM2 inhibitor. All three ovarian tumor cell lines were treated with AMG-232 and showed a dose-dependent reduction in cancer cell viability (Fig. 2a). The two high MDM2-expressing cell lines, OVTOKO and OVMANA, showed relative resistance to treatment with AMG-232. Consistent with previous data, AMG-232 treatment led to activation of the p53 signaling pathway in the cancer cells in a dose-dependent manner which was shown by immunoblotting (Fig. 2b). Consistent with the MDM2-knockdown data, the effect of significant reduction in IL-6 expression was observed with AMG-232 in a dose-dependent manner (Fig. 2b). A dose-dependent increase in PD-L1 expression was observed only in the OVTOKO cell line. To further investigate whether MDM2 blockade may lead to sensitization of tumor cells to T-cell-mediated killing, we pre-treated tumor cells with 0 or 1 µM AMG-232 for 8 hours followed by T-cell co-culture with 0 or 1 µM AMG-232 for another 16 hours. Both cell lines with AMG-232 treatment were found to have nearly double the dead/live tumor cell ratio as compared to untreated controls. The OVTOKO dead/live cell ratio was 3.9:1 (*P* < 0.01) versus 7.5:1 (*P* < 0.01) for untreated versus AMG-232 treated tumor cells, respectively (Fig. 2c). Similarly, the OVMANA dead/live cell ratio was 1.7:1 (*P* = 0.02) versus 3.3:1 (*P* < 0.01) for untreated versus AMG-232 treated tumor cells, respectively (Fig. 2c). Importantly, at 1 µM AMG-232, the dose that was used in the T-cell co-culture system, there was no observed toxicity towards the T-cells (Fig. 2d).

To investigate the effect of AMG-232 in combination with anti-PD-1 treatment (pembrolizumab), OVTOKO and OVMANA cells were co-cultured with T-cells and the dead/live tumor ratio for different conditions was determined (Fig. 2e). The combination of pembrolizumab with AMG-232 in the T-cell plus tumor cell co-culture system demonstrated enhanced T-cell-mediated tumor cell killing of the OVMANA cell line (*P* < 0.05). Despite a similar trend with OVTOKO, the difference was not statistically significant. Pembrolizumab pre-treated T-cells without AMG-232 treatment showed a significant increase in T-cell-mediated tumor killing for OVMANA, but no significant effect was observed for OVTOKO.

## Discussion

Despite recent advances in the field of cancer immunotherapy, the treatment benefit has been limited to a minority of patients with certain cancer types and a significant subset of patients who initially respond eventually relapse due to different mechanisms during the course of treatment^7^. Chemotherapy resistance in tumor cells through the MDM2–p53 loop-dependent and -independent pathways has been reported^8^. However, the role of MDM2 in cancer cells in the context of immunotherapy resistance remains not well-understood. The potential role of MDM2 in T-cell activation was shown in a study demonstrating that stabilization of the E3-ubiquitin ligase MDM2 in T-cells negatively regulates T-cell activation by degradation of the transcription factor NFATc2^9^. Importantly, MDM2 amplification in tumor cells has recently been suggested as a potential biomarker for HPD that is observed in some patients (5–29%) after ICI therapy, suggesting a potential role of MDM2 overexpression in cancer cell immune evasion^3,4^. Moreover, a recent study suggested that MDM2 amplification can predict poor response to ICI therapy in a variety of cancers^5^. These findings reinforce the urgent need to understand the role of MDM2 in cancer immunotherapy as a potentially actionable target that may also reduce the risk of disease hyper-progression following ICI therapy.

Despite encouraging results in a variety of cancers such as melanoma, non-small-cell lung cancer, kidney, and urothelial cancers, single agent ICIs have shown only modest results in ovarian cancers^10^. Among histologic subtypes of ovarian cancer, ovarian clear cell carcinoma is particularly known to be relatively resistant to conventional platinum, or taxane-based chemotherapy and is associated with a poor prognosis^11^. Using wild-type p53-expressing ovarian clear cell carcinoma cell lines in the current study, we demonstrate in a preclinical tumor cell plus T-cell co-culture system that high MDM2-expressing human ovarian cancer cell lines are relatively resistant to T-cell-mediated tumor cell killing. This is in support of recent clinical findings suggesting a potential role of MDM2 amplification or overexpression in immunotherapy resistance or in HPD. We therefore interrogated whether targeting MDM2 may favor sensitizing tumor cells to T-cell-mediated killing. Targeting MDM2-loss in resistant tumor cell lines by siRNA or functional blockade by AMG-232 showed enhanced T-cell-mediated tumor killing versus control treatment regardless of PD-L1 expression changes. Consistent with our observation of changes in PD-L1 with MDM2 blockade, a recent study using another MDM2 inhibitor APG-115 resulted in increased surface expression of PD-L1 on tumor cells^12^. The same study also showed that the MDM2 inhibitor in syngeneic models enhanced anti-tumor immunity in the tumor microenvironment via T-cell proliferation, enhanced CD4 + T-cell activation and increased M1 macrophages. In our study, combination of MDM2 inhibitor AMG-232 with anti-PD-1 treatment (pembrolizumab) enhanced T-cell-mediated tumor killing in the OVMANA ovarian cancer cell line. Despite a similar trend in OVTOKO, the effect was not statistically significant which could be due to increased PD-L1 expression in OVTOKO cell line.

Potential relationships between p53 and immune-checkpoint regulators have been previously identified in cancer cells including but not limited to upregulation of PD-1 and its ligand PD-L1 in a p53-dependent manner in stressful conditions, regulating the expression of miR34. miR34 directly binds to the 3′ untranslated region of the gene encoding PD-L1 and controls signaling-mediated phagocytosis of apoptotic cells through its target, Death Domain1α (DD1α)^13–15^. In addition, the effects of p53 activation in macrophages within the tumor microenvironment to enhance anti-tumor immunity have been reported^16,17^. Among the important inflammatory cytokines, IL-6, that promotes tumorigenesis, was previously shown to downregulate p53 by the enhancement of rRNA transcription^18^. Testing its role in cancer immunotherapy, Ohno et al.^19^ showed that a lack of IL-6 produced in a tumor-bearing host augments induction of anti-tumor effector T-cells and inhibits tumorigenesis in vivo. Moreover, targeting IL-6 sensitized anti-PD-L1 treatment in a colorectal preclinical model highlighting the potential role of IL-6 in cancer immunotherapy^20^. Our findings show that targeting MDM2 attenuates IL-6 expression in tumor cells which may play a crucial role in sensitizing tumor cells to T-cell-mediated killing. Similar to our observation, MDM2 antagonists effectively reduced IL-6 secretion from fibroblasts and the effect was found to be p53-dependent^21^.

In conclusion, our findings demonstrate that MDM2 overexpression or amplification in wild-type p53-expressing ovarian tumor cells likely contribute to tumor cell immune evasion. Specific inhibition of MDM2 in T-cell resistant tumor cells with high MDM2 expression decreases IL-6 expression and enhances T-cell-mediated killing regardless of changes in PD-L1 expression of the tumor cells. The combination of pembrolizumab with AMG-232 in the T-cell plus tumor cell co-culture system demonstrated enhanced T-cell-mediated tumor cell killing and thus holds promise for clinical translation. Future studies can investigate in more detail the importance of co-targeting MDM2 and MDM4. While in vivo studies are warranted to further support our findings, we expect that targeting MDM2 will enhance the efficacy of immunotherapy, in part through effects on cytokines such as IL-6, but potentially other mechanisms that are relevant for immune checkpoint therapy-treated tumors regardless of MDM2 expression. Therefore, our findings provide evidence and a rationale for targeting MDM2 using small-molecule MDM2 antagonist treatment in combination with ICI therapy to overcome drug resistance or address HPD in cancer immunotherapy clinical studies.

## Materials and methods

### Cell lines and culture conditions

The human ovarian clear cell carcinoma cell lines carrying wild-type p53, OVTOKO and OVMANA were kindly provided by Prof. David Huntsman, The University of British Columbia, Vancouver, BC, Canada. Another human ovarian clear cell carcinoma, TOV-21G, and the human T-cell line, TAL-104 (expressing CD2+; CD3+; CD7+; CD8+; CD56+; CD4−; and CD16−) were purchased from ATCC. All cell lines were cultured at 37 °C in RPMI‐1640 containing 10% FBS (except 20% FBS was used for TAL-104), 2 mmol/l glutamine, 100 U/ml penicillin, and 100 µg/ml streptomycin. Recombinant human IL-2 (Miltenyi cat# 130–097744) with a final concentration of 100 units/mL was added to the TAL-104 culture media.

### CellTiter-Glo bioluminescent cell viability assay

Cells were seeded at 3000 cells/well on 96-well black plates and treated as desired. The cells were mixed with a 25-μl or approximately the same amount of CellTiter-Glo reagents (Promega, Madison, WI), following the manufacturer’s protocol. The bioluminescence imaging was measured using a Xenogen IVIS imager.

### Western blot analysis and antibodies

Cell lysates were electrophoresed through 4–12% SDS-PAGE then transferred to PVDF membranes. The primary antibodies indicated in the figures were incubated with the transferred PVDF membranes in blocking buffer at 4 °C overnight, then washed with PBS, followed with anti-mouse-HRP or anti-rabbit-HRP incubation for 1 h at room temperature. Antibody binding was detected with ECL on PVDF using the syngene chemiluminescent western blot imaging system. The following antibodies were used for immunoblotting: anti-p53 (DO-1) and anti-MDM2 (SMP14) from Santa Cruz; anti-PD-L1(E1L3N), anti-PARP and anti-DR5 from Cell Signaling; anti-IL-6 from Abcam; anti-P21 (Ab-1) from EMD Millipore; and anti-Ran from BD bioscience.

### siRNA transfection

Cells were seeded on 12-well plates the day before siRNA transfection. For the siRNA transfection experiments, 10 pmol of TP53 siRNA (Thermal Fisher science, AM51331) or MDM2-siRNA (Ambion) was transfected with lipofectamine RNAiMAx (Invitrogen) as recommended by the manufacturer. At 48 h after transfection, cells were treated as indicated in the figures.

### T-cell co-culture system and microscopic imaging for data analysis

CellTracker™ CMFDA (5-chloromethylfluorescein diacetate) green fluorescent dye (5 µM) or CellTracker™ CMAC (7-amino-4-chloromethylcoumarin) blue fluorescent dye (10 µM) were used to stain and detect living tumor cells or T-cells. Before co-culture with T-cells, culture media was removed and pre-warmed CellTracker™ in “working solution” was added as instructed in the manufacturer’s protocol (Invitrogen, Waltham, MA). The working solution with the CellTracker™ was replaced with fresh media after 30 min of incubation at 37 °C. Green fluorescent tumor cells with or without 1 µM AMG-232 (MedChem Express) pretreatment were co-cultured with or without 25 µg/mL pembrolizumab pre-treated cytotoxic T-cells (TALL-104) with a 2:1 effector-to target cell ratio (E:T) for 12 or 16 h. RPMI-1640 media containing 20% FBS and 100 units/mL IL-2 was used in the co-culture system. For analysis of cell death, 1-µM red fluorescent ethidium homodimer-1 (EthD-1) was added and incubated for another 30 min to detect dead cells (Invitrogen, Waltham, MA). For the quantification of dead/live cells, fluorescence microscopy was used to take images at 10× magnification. The number of red/green/blue colored cells in random fields was counted by two independent investigators and expressed as a dead/live cell ratio. At least 100 cells were evaluated per sample, with three independent replicates.

### Statistical analysis

The statistical significance of differences between groups was determined using the Student’s *t* test. The minimal level of significance was *P* < 0.05. *P < 0.05 and **P < 0.01.

### Impact of findings

Despite encouraging results with immune checkpoint inhibitors (ICIs) in various cancers, immunotherapy resistance and hyper-progression (HPD) limit the success and remain as major challenges. The observation of MDM2 amplification in clinical studies as a potential biomarker for HPD and as a predictive signature for poor response to ICIs suggests a regulatory role for MDM2 in cancer immunotherapy. Our study suggests that cancer cells with overexpression or amplification of MDM2 are resistant to T-cell-mediated killing. MDM2 inhibition suppressed IL-6 and enhanced T-cell-mediated killing ±ICI which provides a rationale for targeting MDM2 to overcome drug resistance including hyper-progression in the context of immune checkpoint therapy.

## Supplementary information

Supp Video Legends

Supp Video 1

Supplemental Material File #1

Supplemental Material File #2

Supplemental Material File #3

Supplemental Material File #4

## References

[1] Yan Y (2018). Combining immune checkpoint inhibitors with conventional cancer therapy. Front. Immunol..

[2] Adashek JJ, Kato S, Ferrara R, Lo Russo G, Kurzrock R (2020). Hyperprogression and immune checkpoint inhibitors: hype or progress?. Oncologist.

[3] Kato S (2017). Hyperprogressors after immunotherapy: analysis of genomic alterations associated with accelerated growth rate. Clin. Cancer Res..

[4] Kamada T (2019). PD-1(+) regulatory T cells amplified by PD-1 blockade promote hyperprogression of cancer. Proc. Natl Acad. Sci. USA.

[5] Fang W (2020). MDM2/4 amplification predicts poor response to immune checkpoint inhibitors: a pan-cancer analysis. ESMO Open.

[6] Anglesio MS (2013). Type-specific cell line models for type-specific ovarian cancer research. PLoS ONE.

[7] Sharma P, Hu-Lieskovan S, Wargo JA, Ribas A (2017). Primary, adaptive, and acquired resistance to cancer immunotherapy. Cell.

[8] Hou H, Sun D, Zhang X (2019). The role of MDM2 amplification and overexpression in therapeutic resistance of malignant tumors. Cancer Cell Int..

[9] Zou Q (2014). USP15 stabilizes MDM2 to mediate cancer-cell survival and inhibit antitumor T cell responses. Nat. Immunol..

[10] Ghisoni, E., Imbimbo, M., Zimmermann, S. & Valabrega, G. Ovarian cancer immunotherapy: turning up the heat. *Int. J. Mol. Sci.*10.3390/ijms20122927 (2019). 10.3390/ijms20122927 PMC6628106 31208030

[11] Itamochi H, Kigawa J, Terakawa N (2008). Mechanisms of chemoresistance and poor prognosis in ovarian clear cell carcinoma. Cancer Sci..

[12] Fang DD (2019). MDM2 inhibitor APG-115 synergizes with PD-1 blockade through enhancing antitumor immunity in the tumor microenvironment. J. Immunother. Cancer.

[13] Munoz-Fontela C, Mandinova A, Aaronson SA, Lee SW (2016). Emerging roles of p53 and other tumour-suppressor genes in immune regulation. Nat. Rev. Immunol..

[14] Cortez, M. A. et al. PDL1 regulation by p53 via miR-34. *J. Natl Cancer Inst.*10.1093/jnci/djv303 (2016). 10.1093/jnci/djv303 PMC4862407 26577528

[15] Yoon KW (2015). Control of signaling-mediated clearance of apoptotic cells by the tumor suppressor p53. Science.

[16] He XY (2015). p53 in the myeloid lineage modulates an inflammatory microenvironment limiting initiation and invasion of intestinal tumors. Cell Rep..

[17] Guo G, Yu M, Xiao W, Celis E, Cui Y (2017). Local activation of p53 in the tumor microenvironment overcomes immune suppression and enhances antitumor immunity. Cancer Res..

[18] Brighenti E (2014). Interleukin 6 downregulates p53 expression and activity by stimulating ribosome biogenesis: a new pathway connecting inflammation to cancer. Oncogene.

[19] Ohno Y (2017). Lack of interleukin-6 in the tumor microenvironment augments type-1 immunity and increases the efficacy of cancer immunotherapy. Cancer Sci..

[20] Li J (2018). Targeting interleukin-6 (IL-6) sensitizes anti-PD-L1 treatment in a colorectal cancer preclinical model. Med. Sci. Monit..

[21] Wiley CD (2018). Small-molecule MDM2 antagonists attenuate the senescence-associated secretory phenotype. Sci. Rep..

