# Correction: AMG-232 sensitizes high MDM2-expressing tumor cells to T-cell-mediated killing

**DOI:** 10.1038/s41420-020-00310-1

**Published:** 2020-08-07

**Authors:** Ilyas Sahin, Shengliang Zhang, Arunasalam Navaraj, Lanlan Zhou, Don Dizon, Howard Safran, Wafik S. El-Deiry

**Affiliations:** 1 Joint Program in Cancer Biology, Brown University and Lifespan Health System, Providence, RI USA; 2 Division of Hematology/Oncology, The Warren Alpert Medical School, Brown University, Providence, RI USA; 3 Department of Pathology & Laboratory Medicine, The Warren Alpert Medical School, Brown University, Providence, RI USA; 4 Cancer Center at Brown University, The Warren Alpert Medical School, Brown University, Providence, RI USA

**Keywords:** Cancer immunotherapy, Drug development

Correction to: *Cell Death Discovery*

10.1038/s41420-020-0292-1 published online 6 July 2020

The original HTML version of this article was updated shortly after publication following a formatting issue which resulted in the abstract heading being omitted. This error has now been corrected in the HTML version of the Article. The PDF version of the Article was correct at the time of publication.

