# Supplementary material for: AMG-232 sensitizes high MDM2-expressing tumor cells to T-cell-mediated killing
Source: Cell Death Discov. 2020 Jul 6;6:57. doi: 10.1038/s41420-020-0292-1 (PMC7338458; doi:10.1038/s41420-020-0292-1)
Supplement: Supplementary file 1 — Supp Video Legends [file 41420_2020_292_MOESM1_ESM.docx]

**Supplementary Video Legends**

**Supplementary Video 1. High MDM2 tumor cells are resistant to T-cell mediated killing**

**(1A)** Example of a video showing T-cell mediated tumor cell killing. The cell-impermeant viability indicator red fluorescence ethidium homodimer-1 (EthD-1) was used to detect dead cells. Tumor cells were cultured overnight in a chamber-slide and T-cells (in suspension) were added to the media with an effector:target (E:T) ratio of 2:1 in the presence of 1 µM ethdium homodimer-1 (EthD-1) for 8 or 12 hours. Images were captured every 10 or 15 minutes with video microscopy for final video (red arrow showing T cell, green arrow showing tumor cell). **(1B)** Under high magnification (32X), OVTOKO (high MDM2) and TOV-21G were cultured overnight and co-cultured with and without T-cells (TALL-104) with an E:T ratio of 2:1 in the presence of EthD-1 for 8 hours. **(1C)** Single tumor cell video of OVTOKO and TOV-21G showing tumor cell interaction with T-cells.

**Supplementary Video 2. Targeting MDM2 by siRNA sensitizes tumor cells to T-cell killing**

MDM2- or control-siRNA transfected OVTOKO and OVMANA tumor cells were examined after T-cell co-culture. T-cells were added to the media at an E:T ratio of 2:1 in the presence of 1 µM ethidium homodimer-1 (EthD-1) for 12 hours and images were captured for the video. Left panel shows OVMANA siRNA control versus MDM2 in the T-cell co-culture system with red fluorescence indicating cell death. Similarly, the right panel shows OVTOKO siRNA control versus MDM2 in the T-cell co-culture system.
