# Supplementary material for: AMG-232 sensitizes high MDM2-expressing tumor cells to T-cell-mediated killing
Source: Cell Death Discov. 2020 Jul 6;6:57. doi: 10.1038/s41420-020-0292-1 (PMC7338458; doi:10.1038/s41420-020-0292-1)
Supplement: Supplementary file 2 — Supp Video 1 [file 41420_2020_292_MOESM2_ESM.pptx]

## Slide 1
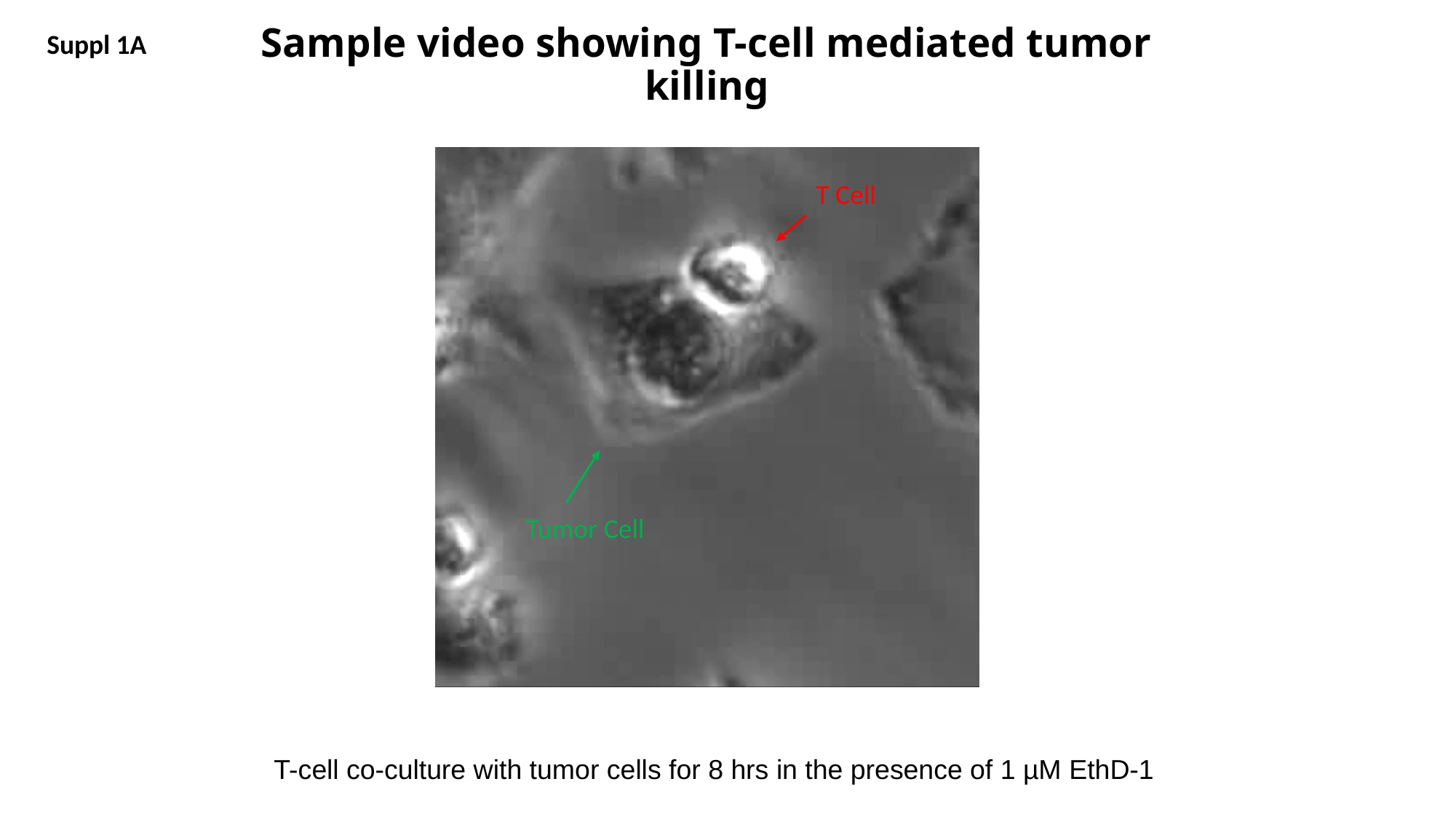

Sample video showing T-cell mediated tumor killing
Suppl 1A
T Cell
Tumor Cell
T-cell co-culture with tumor cells for 8 hrs in the presence of 1 µM EthD-1

## Slide 2
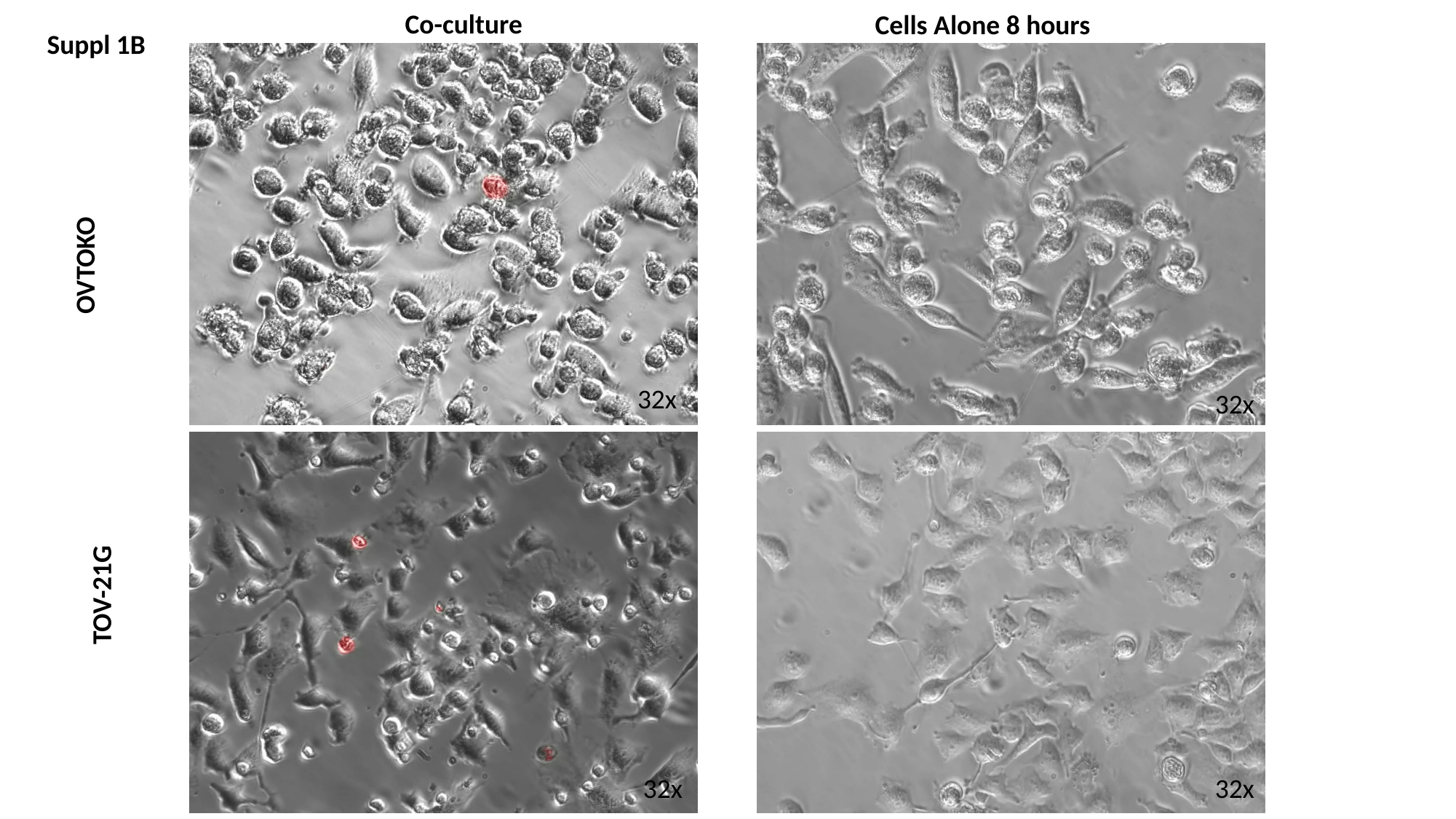

Co-culture
Cells Alone 8 hours
Suppl 1B
OVTOKO
32x
32x
TOV-21G
32x
32x

## Slide 3
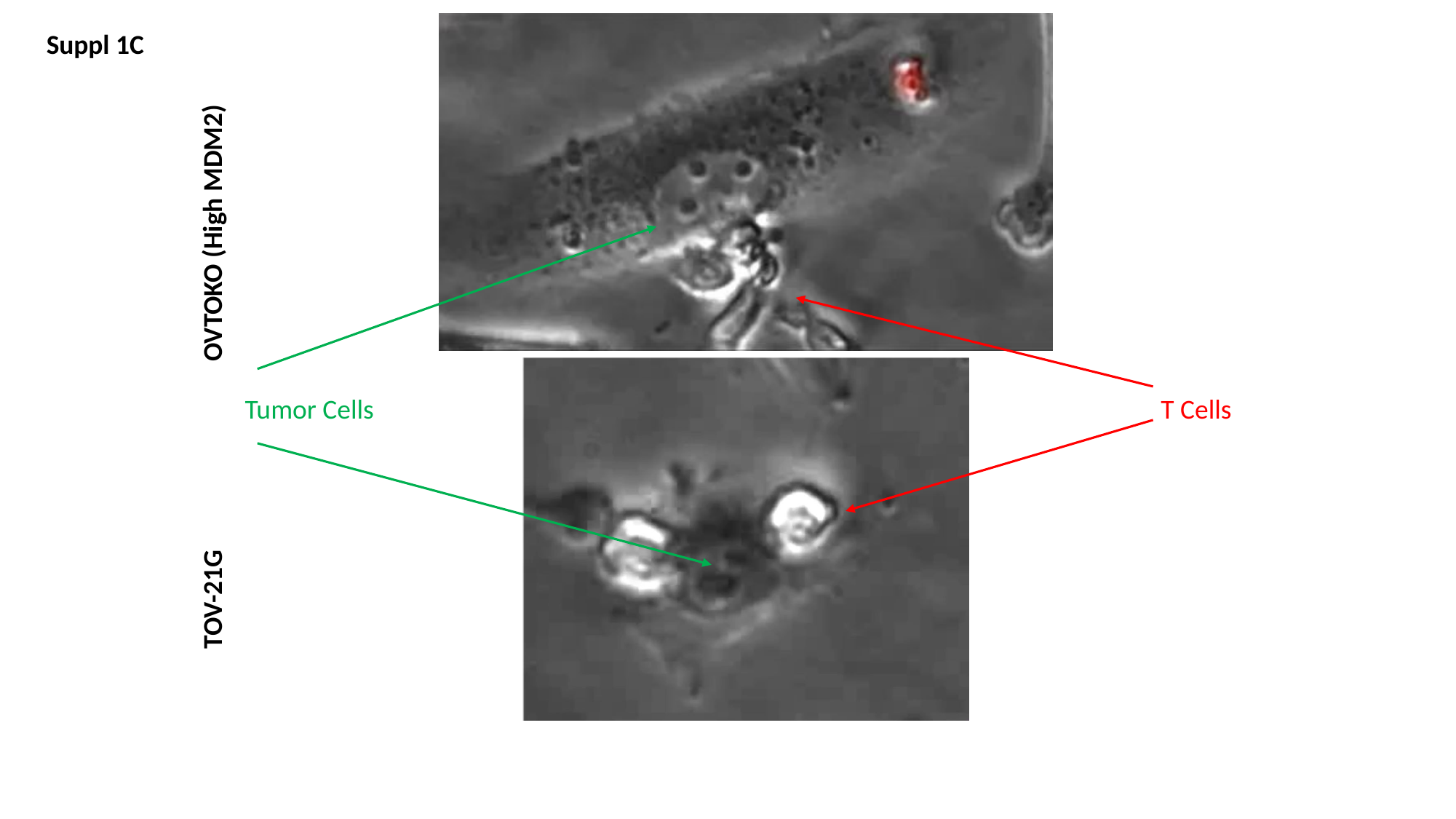

Suppl 1C
OVTOKO (High MDM2)
Tumor Cells
T Cells
TOV-21G
